# By hook or by crook: how and why do compound leaves stay curved during development?

**DOI:** 10.1093/jxb/eraa389

**Published:** 2020-10-22

**Authors:** Yasmine Meroz, Wendy K Silk

**Affiliations:** 1 School of Plant Sciences and Food Security, Tel Aviv University, Tel Aviv, Israel; 2 Department of Land, Air, and Water Resources, University of California, Davis CA, USA

**Keywords:** Curvature, growth kinematics, leaf, morphogenesis, proprioception, quantitative approaches

## Abstract

This article comments on:

**Rivière M, Corre Y, Peaucelle A, Derr J, Douady S**. 2020. The hook shape of growing leaves results from an active regulatory process. Journal of Experimental Botany **71**, 6408–6417.

From the size scale of the DNA molecule to the branches of the largest oak trees, curved and twisted forms are evident in plants and important for biological function. Rivière *et al.* (2020) report that during expansion of compound leaves, the leaf stem (rachis) is maintained curved. The rachis associated with a developing leaflet pair first curves, near the tip, and then straightens farther away from the tip, causing the developing leaf to maintain a hook shape that is displaced from the plant axis while the hook is also maintained a fixed distance from the leaf tip. The authors found this developmental pattern in many species of compound leaves. They characterized it in detail in leaves of the star fruit tree, *Averrhoa carambola*.

When quantitative approaches from physics or engineering are used to study plant form and movement, they often illuminate the mechanics of organ curving (Box 1). Building on a great deal of physics-informed research (e.g. Sharon *et al.*, 2007), recent work includes elucidation of the mechanism of curving in flowers (Woollacott et al., 2019) and bending tropisms of plants (Bastien et al., 2013, 2014; Porat *et al.*, 2020).

Box 1. **Examples of curved plant organs, produced by different mechanisms**

(Left) In broad-leaved seedlings, an apical hook is maintained by gradients in elongation rate within the zone of primary growth (Silk and Erickson, 1978). (Center) In the maize leaf, curvature distal to the primary growth zone is thought to be produced by contraction of developing fibers on the abaxial (lower) leaf surface (Moulia *et al.*, 1994; Hay *et al.*, 2000). (Right) The ruffled edge of the giant kelp blade is produced by faster growth of the edge relative to the interior of the wide, thin blade (Koehl *et al.*, 2008).

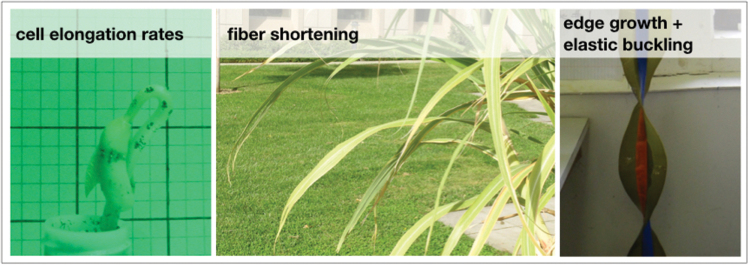

## Quantitative approaches: curvature, and site-specific and tissue-specific characterization

Rivière *et al*. use concepts familiar to engineers but not always part of the botanist’s toolbox. First, they use the mathematical definition of curvature, symbolized κ, which is the rate of change of the local angle θ along a curve (see Box 2). This can be alternatively understood by noting that at every point along the rachis one could fit a circle; the reciprocal of the radius of the fitting circle is the local curvature. Visually we see that a small fitting circle indicates large curvature (equivalent to a significant change in angle) while a region with zero curvature, fit by a circle of infinite radius, is straight (angles do not change). Using the mathematical definition allows the authors to describe the spatial distribution of curvature along the rachis and then to compare this with the spatial pattern of botanical properties including local elongation rates and lignification. This is in contrast to the ‘angle of curvature’ favored by botanists since the time of Darwin. The angle of curvature is the integral of the local curvature κ along the organ, and therefore it is a single global property, analogous to organ elongation rate. The angle of curvature does not allow analysis in terms of underlying physiology or genetics. It is the mathematical definition of local curvature that has permitted understanding of the different ways in which organisms produce curved forms, including the genetic basis for curvature (e.g. Coutand *et al.*, 2009).

Box 2. **Active and passive elements of posture control in plants**

(a) Active posture control in plants: gravitropism and proprioception. Four snapshots from the gravitropic response of Impatiens glandulifera from Pfeffer’s original photographs [Pfeffer]: (1) the initial state, when the plant is placed horizontally; (2) the plant senses the direction of gravity and starts to grow upwards; (3) the plant overshoots the vertical direction; and (4) the organ straightens out and reaches its vertical steady state, and will no longer overshoot. The fact that the plant is able to straighten and does not overshoot again is due to the plant’s ability to sense its own curvature, termed proprioception. The local angle at point *s* at time *t*, θ(*s*,*t*), and the local curvature κ(*s*,*t*) are illustrated here, as described in the main text. The yellow segments in the initial and final snapshots highlight the same tissue element, illustrating tissue-specific characteristics. The equation describing the kinematics of active posture control (Bastien *et al.*, 2013) is made up of three terms: the rate of change of the curvature in time ∂κ/∂*t* is dictated by the graviceptive term βsinθ, and the proprioceptive term κγ (where β and γ are the graviceptive and proprioceptive sensitivities).

(b) Passive elastic response of plants to their self-weight. Plants have mechanical properties and respond passively to forces in the environment such as gravity (as opposed to gravitropism, the active response to gravity). The authors used beam theory to extract the mechanical properties of the rachis, which relates the torque Γ applied on a beam to its flexural rigidity *B*, the observed curvature κ resulting from the ‘drooping’ due to self-weight (denoted K_||_ in Rivière *et al*.), and the intrinsic curvature κ* being the curvature of the beam when no forces are applied (denoted K_0_ in Rivière *et al*).

(c) Model incorporating both active and passive responses. Chelakkot and Mahadvan (2017) relate the intrinsic curvature of the organ κ, which is actively regulated, to the actually observed curvature κ* due to self-weight. By assuming that the time scale of active regulation is significantly larger than the time scale of mechanical responses, they can incorporate this relationship within the description of active regulation in panel (a).

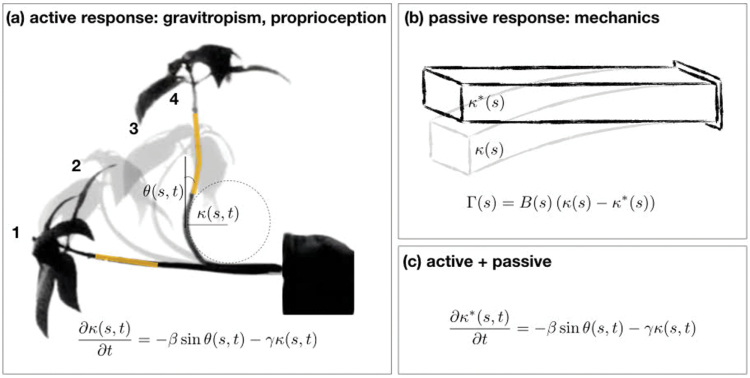

A second subtlety to this study is the attention paid to the distinction between the time courses of local (site-specific) curvature and tissue-specific curvature. A site-specific view at 2 cm from the tip of the leaf shows that the curvature decreases slowly but remains high during 6 d of leaf development (Rivière *et al*., fig. 3, Days 10–15). This observation does not give us a sense of the fate of an individual rachis segment. For instance, the rachis supporting the third pair of leaflets experiences complete straightening over a 1 d period, as it is displaced over a few centimeters of distance during its development (Rivière *et al*., fig. 3, comparing Day 12 with Day 13). This notion is illustrated in Box 2, where a yellow-shaded, growing stem segment is shown at two different times and locations during gravitropism. Although botanists trained in morphogenesis often appreciate the site–tissue duality (quantified in Green, 1976 and Silk and Erickson, 1979), those trained in physiology sometimes do not. It is only by following developing tissue elements through space and time, as Rivière *et al*. have described, that one can understand the leaf morphogenesis. This paper shows that the maintenance of a stationary hook from a parade of changing tissue elements, first described for hypocotyls, is quite common in plant development.

## Generalizing the model for proprioception

The authors raise intriguing possibilities for a generalized model of proprioception. A recent model (Chelakkot and Mahadevan, 2017) suggests that plant posture control is generally governed by a combination of passive physical processes and active biological processes (Box 2). Physically, a plant organ has mechanical properties that dictate how it will bend passively in response to an external force, such as gravity (e.g. Silk *et al*, 1982). For the sake of clarity, in what follows we adopt the nomenclature used in Chelakkot and Mahadevan (2017), while noting the equivalent notation used in Rivière *et al.* (2020). Given the mechanical properties of the organ, beam theory relates the applied moment of force to the difference between the observed curvature κ (denoted K_||_ in Rivière *et al.*, 2020) and the intrinsic curvature κ*, where no forces are applied (denoted K_0_ in Rivière *et al.*, 2020) as shown in panel b in Box 2.

Chelakkot and Mahadevan combine the equations describing the active and passive processes, and the resulting equations are similar to those describing active regulation alone. In their model the intrinsic curvature of the organ, κ*, is actively regulated by gravitropism and proprioception, where proprioception acts on the actual, observed curvature κ alone (see panel c in Box 2). This means that the proprioceptive term γκ disappears when the observed curvature is zero (i.e. the organ is straight), which implies that the desired, or target curvature is zero.

However, this does not reflect the findings of Rivière *et al*., where the desired curvature of the tip of the rachis has a non-zero value K_0_. From this, we can deduce that in the case of the rachis, the target curvature is K_0_, and the proprioceptive term should vanish when the observed curvature κ reaches this curvature. In other words, the proprioceptive term should take the form γ(κ–K_0_). Rivière *et al*. also show that the hook shape persists even with the disruption of graviception, which tells us that this target curvature is somehow pre-determined, allowing the intrinsic curvature to change accordingly. This is a subtle point which suggests that the term ‘intrinsic’ curvature refers to slightly different things in the elasticity model by Chelakkot and Mahadevan and in the work by Rivière *et al*. We suggest that Riviere’s term K_0_ might be termed ‘target’ curvature, since it is pre-determined, distinguishing it from Chelakkot’s intrinsic curvature κ*. This observation could then be formulated in a more general model: ∂κ*/∂*t*= –βsin(θ)–γ(κ–K_0_). This is particularly interesting in light of the fact that Rivière *et al*. show that the target curvature persists also without graviception. Hence, the connection between the target curvature K_0_ and the actively regulated intrinsic curvature κ* is not clear, not even at the conceptual level and certainly not at the biological level.

Another interesting observation made in Rivière *et al*. is the fact that the target curvature K_0_ is not constant along the rachis, but rather peaks within the growth zone, and then decreases to zero near the end of the growth zone. It should be noted that the straightening is non-trivial, since any small amount of curvature leaving the growth zone would be accumulated over time and would lead to a large angle outside the growth zone. The fact that the rachis seems perfectly straight outside of the growth zone suggests that regulation is extraordinarily precise.

## Future work on function and mechanism of compound leaf curving

This paper raises interesting questions about plant function. Why does developing leaf tissue bother to curve and then straighten? One effect pointed out by the authors is that the leaf tip always points straight down. Does this protect the tip from incident radiation? Furthermore, leaflets in a particular developmental stage are always borne by a horizontal segment of rachis. Does this facilitate photosynthesis at a particular stage? Another effect is reduction of the moment arm on rachis tissue near the shoot axis during leaf development. Is this important for mechanical stability?

Physiologists will be intrigued by the supplementary figures. The beautiful time-lapse movie shows a diurnal pattern of curvature synchronized with leaflet movement. The hook opens slightly during the illumination periods when the leaflets move regularly from vertical to horizontal. What kinds of transport processes regulate these daily rhythms?

Furthermore, the supplementary figs S3, localization of the growth zone, and S5, spatial pattern of lignification, pose essential questions of mechanism. Is the active straightening process regulated by a reversal in the primary growth rate gradient across the rachis? (That is, does the adaxial side grow faster during the hook induction, while the abaxial side grows faster during the unfurling?) Or could fiber development be the main regulating process for unfurling? Getting higher resolution on the growth analysis could answer some of these questions. The suggestion of fiber development as the driver of the straightening process is also intriguing, since it would imply two biologically distinct processes of active regulation (differential growth and adaxial fiber contraction), both acting on the observed curvature but with different target curvatures. The notion of coupled processes of active regulation remains to be further investigated.
